# An empirical study on the moderating effect of environmental audit on the impact of economic development and ecological environment

**DOI:** 10.3389/fpsyg.2022.1047517

**Published:** 2022-11-09

**Authors:** Mingliang Xiong

**Affiliations:** School of Economics Management, Huizhou University, Huizhou, Guangdong, China

**Keywords:** moderating effect, questionnaire survey, environmental auditing, economic development, ecological environment

## Abstract

Environmental audit is a new thing. As an important part of national audit, it is an important environmental governance supervision and control mechanism in China, but what is the macro environmental governance effect of environmental audit? Can environmental audit promote regional pollution control? Does environmental audit play a role in regulating the effect of environmental pollution control on the basis of economic development? The research on these issues has great practical and theoretical significance. Through questionnaire, 217 valid data were finally obtained as research samples. Using correlation analysis, partial correlation analysis, full regression, and stepwise regression analysis, on the basis of testing the explanatory power of economic development and environmental audit on ecological environment quality, the paper further tested the impact of the interactive multiplier of environmental audit and economic development on ecological environment quality. The study found that: not only do economic development and environmental auditing each have a significant positive correlation on the quality of the ecological environment; moreover, economic development and environmental auditing interact with each other to influence the ecological environment, and the degree of correlation and influence of environmental auditing on the ecological environment is greater than the degree of correlation and influence of economic development on the ecological environment. Environmental auditing has a significant positive moderating effect on economic development in the process of improving ecological and environmental quality, and on the basis of economic development, environmental auditing by the government has a more significant effect on the improvement of ecological and environmental quality. Studying this problem is not only conducive to guiding the government audit department to take on the role in the ecological environment governance, but also to more effectively control the environmental pollution problem, realizing the cooperation and mutual penetration of the role of government environmental audit in economic development and ecological environment protection policies. It provides reference for future empirical research using micro survey data, and also provides policy suggestions for economic construction, environmental audit, and ecological environment governance in various regions of China, and provides empirical evidence support for contingency theory.

## Introduction

High-quality development lies in the harmonious integration of “economic-ecological-social” benefits. The relationship between economic development and the ecological environment is an eternal proposition that needs to be balanced in the development of all countries in the world. The ecological environment is the basis for human survival and development, but the process of economic development is often at the expense of the environment. In recent years, the issue of ecological protection and environmental governance has been a hot topic of global concern. After more than 40 years of reform and opening up, China’s economic development has soared to global prominence with brilliant results. But at the same time, there has also been massive consumption of energy resources and serious damage to the ecological environment, some of which is alarming to the eye. Therefore, in order to achieve high-quality development, the importance of protecting the ecological environment and managing ecological pollution cannot be overstated. As an important type of national audit, environmental audit is an important environmental protection tool and a vital “immune system” (Liu, 2015) in the national economic governance system. It is a veritable guardian of the environment, playing a unique role of monitoring, early warning, revealing, and correcting in coordinating the process of dealing with “economic, ecological, and social” conflicts.

Mishan (1967) argues that economic development and the ecological environment are contradictory, and that while economic development enriches the material life of local people, it also degrades the local ecological environment, which in turn constrains further development of the local economy. Parry (1998) found that economic growth can promote the improvement of environmental regulation, and there is no restriction on the improvement limit of ecological environment quality. Shi et al. (2020) found that economic development and the ecological environment are in an intermediate stage of coupling and coordination, with significant spatial and temporal variability, using Chinese regions as a sample. Wang and Liu (2017)constructed an empirical spatial joint cubic equation model for the relationship between economic development, urbanization, and environmental pollution based on 253 regional-level data from 2003 to 2014 across China, and found that economic development and environmental pollution influence each other, and the EKC environmental Kuznets curve is a two-way inverted “U” shape. Zhao et al. (2019) studied the mechanism of economic growth on the improvement of ecological and environmental quality using a spatial panel model based on data from 30 provinces in China between 2003 and 2016, and found that economic development promoted technological progress, which in turn enhanced government environmental governance, thus significantly improved the local ecological and environmental conditions.

In recent years, domestic and international scholars have studied the effects of environmental auditing and environmental governance. Stafford (2006) argues that environmental auditing is an internal review of the results of environmental performance-related behaviors, used to verify compliance with environmental regulations and the effectiveness of environmental management systems. Prajogo et al. (2016) argue that the motivation of environmental auditing is often based on environmental performance improvement to obtain external accreditation and improve public satisfaction in society. Cai et al. (2021) found that audits can identify monitoring priorities, and in areas with high levels of environmental pollution, audits are more intense and government environmental governance is more efficient. In addition, environmental audits can also play a micro governance environmental effect (Yu et al., 2020), improve the level and quality of corporate environmental responsibility information disclosure (Ding and Hu, 2022), and improve corporate environmental protection investment (Cai et al., 2021), which suggests that environmental audits can effectively play a social responsibility regulatory role. Empirical studies of environmental audits have also shown that conducting environmental audits can indeed improve environmental performance; Chen et al. (2022) summarized the findings of the 10th worldwide environmental audit published by the Working Group on Environmental Auditing (WGEA) and concluded that environmental auditing can both protect economic development and be an important part of achieving the “3060” double carbon goal^1^. As a result, countries around the world are placing increasing emphasis on the use of environmental audits to evaluate the “economy-ecology” relationship.

However, it can be seen that there are many studies on the relationship between economic development level or government environmental audit and ecological environment quality in the existing literature, but few studies neither integrate the three, nor do they find that government environmental audit is studied as a regulatory variable, thus separating the relationship between government environmental audit and economic development level and ecological environment quality, ignoring the overall effect of the three. As we all know, the governance of the ecological environment has risen to the national strategic level. With the proposal of the “double carbon” goal, the protection and governance of the ecological environment is a major systematic project. The complexity and externality of environmental pollution cannot effectively solve environmental problems based on unipolar forces. In theory, the regions with stronger economic vitality pay more attention to solving the problem of disharmony between economic development and population, resources, environment, and ecology, and also pay more attention to playing the role of environmental audit in pollution prevention. By improving audit technology and strengthening audit power, the audit effect is more obvious, and the performance of environmental governance is more significant (Zhao and Rong, 2022). Wu and Guo (2019) also believed that government environmental audit not only directly affects the construction of the ecological environment, but also indirectly affects the protection and governance of the ecological environment through economic development, so as to further improve the quality of the ecological environment. In view of this, this paper selects 21 regional government audit departments, ecological and environmental departments, natural resources departments, and financial departments in Guangdong Province to conduct a questionnaire survey, and will collect micro-survey data as a research sample to explore the relationship between economic development, environmental audit, and ecological and environmental quality, and further test the moderating effect of environmental audit in the process of regional ecological and environmental quality improvement. It will not only inspects the contingency theory of relevant audit monitoring and management for government audit departments, but also provides a reference for future decisions on economic construction, environmental audits, and ecological management in various regions.

The marginal contribution of this study is as follows: First, compared with the existing research, based on the regulatory effect of environmental audit, this paper tests that environmental audit as a regulatory variable will enhance the positive relationship between economic development and ecological environment quality, which enriches the theoretical research of environmental audit; Second, in previous studies, when examining the influencing factors of ecological environment pollution control performance, they often used macro empirical data, while ignoring micro survey data. This study specifically incorporated micro questionnaire data for empirical testing, expanding the research vision of data; Third, this paper selects 21 regions in Guangdong Province as the research object, which can provide direct evidence support for the government to carry out environmental audit to more effectively govern environmental problems and help achieve the goal of “double carbon.”

### Limitations of this study

Although the paper is objective, unified and extensive in the questionnaire survey, the results are more accurate. However, the questionnaire is only for Guangdong Province after all, and there are certain limitations in the research area. Whether the research conclusions are valid in other provinces in China still needs to be further verified. In addition, the design of the questions and answers of the questionnaire will largely influence the user’s answers, which makes the respondents’ answers more limited and may omit some information, making the research design a little biased.

## Theoretical analysis and research hypothesis

### Mechanistic analysis and research hypothesis of the impact of economic development on the ecological environment

The impact of economic development on ecological environment quality is a combination of indirect and direct effects, and is the result of a game between positive and negative impacts (Zhang and Liang, 2020). There are three main academic findings on the impact of economic development on the ecological environment: the first view is that economic development has a facilitating effect on the ecological environment (Zhao et al., 2019). The second view is that economic development has a hindering effect on the ecological environment (Li and Wei, 2010). A third view is that the impact of economic development on the ecological environment is non-linear (Peng et al., 2020; Ren et al., 2021).This variation in findings may be related to different countries, different stages of development and policy implementation. From the perspective of China’s national conditions, in the early stage of economic development, rapid economic growth often takes the cost of sacrificing the environment. At the same time, with the rapid development of technology and the enhancement of the government’s environmental awareness, after the rapid economic development, the effect, strength, and funds of environmental governance will also increase, thereby significantly improving the environmental and ecological quality (Wang and Liu, 2017).

As far as Guangdong Province is concerned, in the early stage of economic development, the industrial structure was dominated by the secondary industry, presenting a state of low technology enterprises and low added value. At the same time, the shortage of resources and the sacrifice of the ecological environment restricted the economic development (Zhu and Chen, 2011). Wu and Wu (2011) conducted an empirical study on the relationship between economic development and environmental pollution in the three regions based on the data of Guangfo and Zhaozhou Economic Circle from 1996 to 2008. They found that the impact of economic development in different regions on environmental pollution was significantly different in terms of pollution degree, types of pollutant emissions and emissions. Therefore, previous studies have not reached a consensus conclusion on the relationship between economic development and ecological environment. Based on the above mechanism analysis, the following research hypothesis is proposed.

*Hypothesis 1*: There is a significant positive correlation between economic development and ecological environment, and economic development can significantly contribute to the improvement of ecological environment quality.

### Mechanistic analysis and research hypothesis of the impact of government environmental auditing on the ecological environment

Environmental audit, as a national audit, is an institutional arrangement in the national governance system and can exert governance effects at the consciousness and institutional levels (Liu, 2015). In other words, environmental audits can promote the implementation of environmental protection regulations and establish environmental and ecological awareness in the whole society, for example, Cai et al. (2019)found that regions where environmental audits were implemented had higher awareness of social responsibility and higher levels and quality of corporate environmental responsibility information disclosure. This suggests that government environmental audits enable the government and the public to work together to build an ecological civilization, to comply with environmental guidelines, and to “prevent” ecological degradation. In addition, state audits can contribute to the improvement of systems, institutions, and mechanisms to counteract various “diseases” in economic and social operations (Liu, 2015). The process of implementing environmental audits is also a process of improving, perfecting, and standardizing the relevant environmental protection system. Through the implementation of environmental audits, the unreasonable aspects and loopholes in the rules and regulations can be remedied to regulate and guide the behavior of government departments and officials, so that the deterrent effect of state audits can be brought into play and the ecological and environmental damage can be counteracted. According to the above analysis, environmental audit evaluation can play a role in promoting the construction, protection, and governance of the ecological environment and maintaining the harmonious development of ecological civilization through the functions of prevention, defense, revealing, handling penalties, and making recommendations. Based on the above theoretical analysis, the following research hypothesis is proposed.

*Hypothesis 2*: There is a significant positive correlation between environmental auditing and the ecological environment, and environmental auditing helps to significantly improve the quality of the ecological environment.

### Mechanistic analysis and hypothesis of the impact of environmental auditing on the relationship between economic development and ecological environment quality

According to the public fiduciary responsibility theory, environmental problems are external diseconomies under market economy conditions, and therefore solving environmental problems is transformed into an economic responsibility, with the government being entrusted by the public with the primary responsibility for solving environmental problems (Zhou, 2011). In fact, the fundamental objective of the creation of state audits is to facilitate the full implementation of the government’s public fiduciary economic responsibility. In other words, environmental audits can lead the state to fulfil its public fiduciary economic responsibility and solve environmental problems in economic development. At present, environmental audits mainly focus on major resource projects, major environmental issues, and major capital investment (Wang and Zhang, 2019), which involve projects of significant nature and amount. Both major resource projects and major capital investment are important projects to promote China’s economic development, to prevent power abuse and improve the efficiency of capital use through economic responsibility audits, and to meet environmental protection needs and improve economic profitability through environmental audits. Environmental auditing can promote sustainable economic development and achieve a green economy by monitoring the abuse of power and the use of funds, which in turn can achieve the function of environmental protection and reduce ecological damage (Liu, 1997). At the same time, the government environmental audit covers a wide range of issues. Environmental protection problems exist in all walks of life. The national resource and environmental protection policies also involve finance, credit, industrial upgrading, and investment and other economic development fields. Therefore, the environmental audit evaluation not only directly affects the construction of the local ecological environment, but also indirectly affects the governance and protection of the local ecological environment through economic development (Wang, 2011; Wu and Guo, 2019). In other words, government environmental audit may play a more active role in monitoring and controlling the impact of economic development on regional ecological environment. Based on the above mechanistic analysis, the following research hypotheses are proposed.

*Hypothesis 3*: Economic development and environmental auditing interact and jointly influence the ecological environment, and the degree of correlation and influence of environmental auditing on the ecological environment are greater than the degree of correlation and influence of economic development on the ecological environment.

*Hypothesis 4*: Government environmental auditing has a positive moderating effect on economic development in improving the quality of the ecological environment, and on the basis of economic development, government environmental auditing has a more significant effect on improving the quality of the ecological environment.

## Research design

### Questionnaire structure design

The basic structure of the questionnaire adopts the questionnaire design method suggested by Davis (1989), in which all the questions of an indicator to be measured are grouped together to facilitate the coherence of the thinking of the respondents when answering the questionnaire. The questionnaire design in this paper, consists of two main parts: the first part is the control variables, i.e., the individual characteristics of the respondents, mainly including gender, nature of work unit, education, and profession, etc., with a total of four question items; the second part focuses closely on the three main variables of economic development level, environmental audit, and ecological environment quality for question design.

In order to ensure the overall reliability and validity of the questionnaire, 62 experts in the fields of environment, audit and finance were asked to pre-survey and test the reliability and validity of the pre-survey questionnaire, which resulted in a formal questionnaire with five questions for each variable, 15 compulsory questions, and one open-ended question. Sample questions include “How does your family’s economic situation compare with that of the past 10 years,” “What do you think of the current material standard of living,” “How effective is the environmental audit work in your area in reducing pollution in enterprises?” “How satisfied you are with the construction of ecological civilization and the improvement of ecological environment in your area,” etc. In this paper, all the compulsory questions of the three main variables in the questionnaire were judged using a five-point Likert-type scale, with scores ranging from 1 to 5, indicating very poor, poor, fair, good, and very good in that order, with higher scores being more desirable. The structure and reliability of the questionnaire are shown in Table 1.

**Table 1:** Questionnaire structure and reliability and validity test.

Variables and codes	Topic options Number and code	Type	Reliability Cronbach’s α	Validity Kaiser-Meyer-Olkin	Role
Individual Respondent Characteristics	4	Non-scale	0.874(Overall)	0.822(Overall)	Control variables
Economic development (*“rjgdp”*)	*R1*, *R2*, *R3*, *R4*, and *R5*	scale	0.849	0.744	Regression analysis
Environmental Audit (*“eaud”*)	*A1*, *A2*, *A3*, *A4*, and *A5*	scale	0.928	0.879	Regression analysis
Ecological environment quality (*“eqi”*)	*E1, E2, E3, E4,* and *E5*	scale	0.919	0.842	Regression analysis

### Questionnaire distribution and return

In order to control for possible effects of differences in the variables at the regional and industry levels where the sample is located, the research sample for this paper was drawn from 21 regional government departments in Guangdong Province, such as audit, environmental protection, natural resources, and finance, which are closely related to this topic, and the survey period was from March 1 to March 20, 2022. In order to make the sample more representative and scientific, the inclusion and exclusion criteria of the dataset in this study are as follows.

#### The inclusion criteria of the dataset

The samples are all permanent residents with Guangdong household registration on December 31, 2021; the samples were collected from 21 regions in Guangdong Province, including Guangzhou, Shenzhen, Foshan, Dongguan, Huizhou, Zhaoqing, Zhongshan, Maoming, Zhanjiang, Jiangmen, Zhuhai, Shantou, Chaozhou, Jieyang, Shanwei, Shaoguan, Qingyuan, Meizhou, Heyuan, Yangjiang, and Yunfu. The sample is from government audit, environmental protection, natural resources, financial departments, typical enterprises, and institutions who have worked for 3 years or more, and some community residents’ representatives. According to the number of permanent residents in each region by the end of 2021, the total number of samples is determined by the probability sampling method combining stratified sampling and systematic sampling.

#### The exclusion criteria of the dataset

Non Guangdong household registration; among those who have worked in government audit, environmental protection, natural resources, financial departments, typical enterprises, and institutions for 3 years or more, those who fail the year-end assessment; the community representatives are under 25 years old.

In the questionnaire guide, the respondent was told that “This questionnaire is anonymous, any information you have provided is used for academic analysis only, does not involve individual discussions, and the content is absolutely confidential. Please fill in the answer truthfully to ensure the authenticity and scientific nature of the results.” The questionnaires were distributed and collected by the researcher himself, with explanations given prior to distribution. In addition, based on the following points, the sample will be used and participants’ answers will be excluded from the analysis.

Fill in incomplete or unclear questionnaires; questionnaires that take too long to answer (more than 8 min); questionnaires with too short answer time (less than 2 min); and an questionnaire with multiple options appears.

A total of 246 questionnaires were distributed in this study, through the above exclusion, we finally obtained 217 valid questionnaires, with an effective rate of 92.7%, and their individual structural characteristics are shown in Table 2.

**Table 2:** Analysis of individual structural characteristics of the questionnaire sample.

Item	Sample distribution	Number of samples (pcs)	Percentage (%)	Total (pcs)
Gender	Male	94	43.3	217
	Female	123	56.7	
Nature of work unit	Government audit department	90	41.5	217
	Government environmental protection department	43	19.8	
	Natural resources department	27	12.4	
	Finance department	22	10.1	
	Enterprises and institutions	19	8.8	
	Other	16	7.4	
Education	High school and above	2	0.9	217
	College	32	14.7	
	Bachelor’s degree	143	65.9	
	Master’s degree and above	40	18.4	
major	Accounting, auditing and other management	75	34.6	217
	Environmental protection, such as ecology and environment	30	13.8	
	Finance, economics, etc.	67	30.9	
	Other majors	45	20.7	

## Empirical analysis of survey data

### Descriptive statistics and correlation analysis

Table 3 below reflects the descriptive statistics and correlation analysis of the overall survey values of *“rjgdp*,*” “eaud*,*”* and *“eqi,”* and by two-tailed test, it is found that the correlation coefficients between *“rjgdp”* and *“eaud,” “eaud”* and *“eqi*,*”* and *“rjgdp”* and *“eqi”* are 0.803, 0.856, and 0.772, respectively, showing a significant positive correlation at the 0.01 level, initially indicating that “*rjgdp*” and *“eaud”* have a facilitating effect on the improvement of *“eqi,”* but the degree of influence between the variables cannot be judged based on the degree of correlation alone, and the experience of causality must be determined by regression analysis.

**Table 3:** Correlation analysis of the overall data for the three variables of economic development, environmental audit, and ecology.

Variable	Number of samples	Average value	Standard Deviation (SD)	*rjgdp*	*eaud*	*eqi*
*rjgdp*	217	3.86	0.7705	1		
*eaud*	217	3.92	0.7332	0.803^***^	1	
*eqi*	217	3.94	0.7017	0.772^***^	0.856^***^	1

^***^Significant correlation at the 0.01 level (two-tailed).

**Figure 1:**
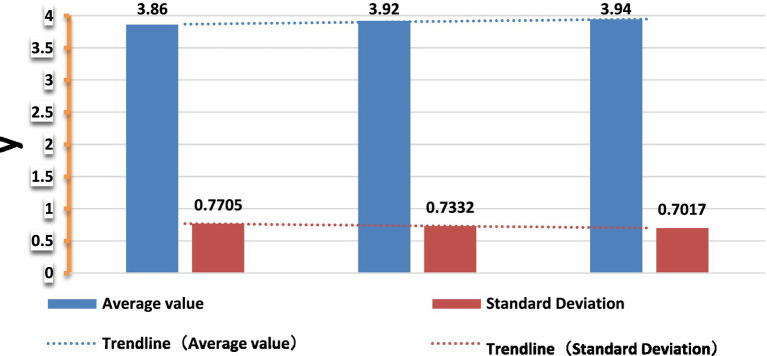
Means and standard deviations of the three variables of economic development, environmental audit, and ecological environment.

As can be seen from Table 3, when evaluating the current level of economic development, the status of environmental auditing and the basic status of ecological and environmental quality in Guangdong Province, the mean scores are relatively high, all of them are above 3.5 (median 3), which indicates that the respondents are satisfied with the current level of economic development, the status of environmental auditing, and the overall evaluation of ecological and environmental quality in Guangdong Province. Figure 1 shows the mean and standard deviation of the three variables of economic development, environmental audit, and ecological environment in Guangdong Province. The figure shows that the average score of economic development level, environmental audit status, and ecological environment quality in Guangdong Province is above 3.5. The score is high and the standard deviation is small, indicating that the interviewees have a high evaluation of Guangdong Province. The more they agree with Guangdong Provincial Government in terms of economic development, environmental audit, and ecological environment governance. Especially, the respondents think that the ecological and environmental quality has been greatly improved through vigorously carrying out environmental audits and focusing on environmental pollution control, which is also in line with the actual situation of Guangdong Province.

### Partial correlation analysis

In the multivariate correlation analysis, due to the influence of other variables, the correlation coefficient of two variables only reflects the nature of the two variables on the surface, often does not truly reflect the degree of linear correlation between variables, and may even give people the illusion of correlation. Therefore, in some cases, a simple two variable correlation coefficient is not an essential statistic to describe the correlation. When other variables are fixed, that is, after they are controlled, the correlation between any two given variables is called partial correlation coefficient. Partial correlation analysis, also known as net correlation analysis, is used to analyze the degree of linear correlation between two variables under the control of the linear influence of other variables. The tool used is partial correlation coefficient. Partial correlation analysis is used to calculate the partial correlation coefficient between variables, so as to more accurately judge the correlation between variables and the degree of correlation. In view of this, this study uses partial correlation to analyze the correlation and degree between economic development, environmental audit and ecological environment.

1. Controlled for the partial correlation analysis of “*rjgdp*” variables, as shown in Table 4.
2. Controlled for biased correlation analysis of *“eaud”* variables, as shown in Table 5.

**Table 4:** Partial correlation analysis of economic development on environmental audit and ecological environment.

Control Variable	Relevant variable	Item	*eaud*	*eqi*
*rjgdp*	*eaud*	Correlation	1.000	0.622
		Significance (two-tailed)	.	0.000
		(number of) Degrees of freedom (physics and statistics)	0	214
	*eqi*	Correlation	0.622	1.000
		Significance (two-tailed)	0.000	.
		(number of) Degrees of freedom (physics and statistics)	214	0

**Table 5:** Partial correlation analysis of environmental audit on economic development and ecological environment.

Control Variable	Relevant variable	Item	rjgdp	eqi
*eaud*	*rjgdp*	Correlation	1.000	0.275
		Significance (two-tailed)	.	0.000
		(number of) Degrees of freedom (physics and statistics)	0	214
	*eqi*	Correlation	0.275	1.000
		Significance (two-tailed)	0.000	.
		(number of) Degrees of freedom (physics and statistics)	214	0

The results of the partial correlation analysis in Tables 4, 5 above show that when “*rjgdp*” is controlled for *“eaud”* and *“eqi,”* the positive correlation between *“eaud”* and *“eqi”* decreases from *r* = 0.856 to *r* = 0.622, with a reduction of 0.234 in the correlation coefficient; when *“eaud”* is controlled for *“rjgdp”* and *“eqi,”* the positive correlation between “*rjgdp*” and *“eqi”* also decreases from *r* = 0.772 to *r* = 0.275, with a surprising reduction of 0.497 in the correlation coefficient. This indicates that economic development and environmental auditing interact with each other and jointly influence the improvement of ecological and environmental quality; on the other hand, it also tentatively confirms that environmental auditing has a greater effect on the improvement of ecological and environmental quality, which tentatively verifies hypothesis 3. However, the partial correlation analysis only shows the correlation between variables and the degree of correlation, but does not determine whether there is a real causality between variables.

### Full regression analysis

In the regression analysis of data, attention should be paid to whether there are multicollinearity and autocorrelation problems. The multiple collinearity of measure is tested by variance inflation factor (VIF), and the autocorrelation of measure is tested by Debbie Watson test (D-W).

Variance inflation factor (VIF) is a measure of the severity of multiple (multiple) collinearity in multiple linear regression models. It represents the ratio between the variance of the regression coefficient estimator and the variance when the independent variables are not linearly correlated. Generally, if the VIF is greater than 10, it is considered that it has a high multicollinearity and the model does not meet the requirements.

Durbin Watson (D-W) is a very effective method to test whether the error items in the model have autocorrelation. The parameter of this test is *D*, and its value range is (0,4). *D* = 2 indicates that residuals are independent; *D* < 2 indicates positive correlation between residuals; *D* > 2 shows that the residuals are negatively correlated. According to experience, when the *D* value is between 1.5 and 2.5, there is no significant autocorrelation problem, and the model is effective (Shen, 2005). In this study, VIF and D-W tests are used to measure the collinearity of survey data.

According to the results of the full regression analysis in Table 6, the “*rjgdp*” and *“eaud”* regression coefficients and constant terms reached significance at the 0.01 level and had a direct effect on *“eqi,”* being introduced into the regression equation. In the regression results, the Fr value is 325.026,significant at the 0.01 level, indicating a very good regression effect, and the correlation coefficient is equal to 0.867, indicating a strong correlation between the independent and dependent variables. *R*^2^ is equal to 0.752, indicating that the independent variable, environmental audit, explains 75.2% of the variability of the dependent variable. The variance inflation factor test (VIF = 2.813) and the Durbin-Watson test (D-W = 1.786) index test, both within the standard range, indicate that there is no autocorrelation between the residuals of the independent variables and there is no problem of multicollinearity. In particular, the regression results of *“eaud”* on *“eqi”* show that the significance level is 1%, the regression system B = 0.635, and *t* value = 11.63. The regression results of “*rjgdp*” on *“eqi”* show that although the significance level is also 1%, the regression system B = 0.218, and *t* value = 4.19, it is obviously inferior to the former. Combining the above information, analysis with the full regression method can reveal that both economic development and environmental auditing have a significant causal influence on ecological environment. Moreover, the correlation and causal impact of environmental audit on the ecological environment are greater than the correlation and causal impact of economic development on the ecological environment. The above analysis further supports the verification of Hypotheses 1–3.

**Table 6:** Table of results of full regression analysis of economic development and environmental audit on ecological environmental.

Regression variable	*R*	*R^2^*	*R^2^* (Adjusted)	*F_r_*	*B*	*B* (Standard)	*t*	VIF	D-W
*eqi*	0.867(a)	0.752	0.750	325.026^***^				2.813	1.786
Constant term					0.609		4.583^***^		
*rjgdp*					0.218	0.239	4.190^***^		
*eaud*					0.635	0.664	11.630^***^		

^***^Significant correlation at the 0.01 level (two-tailed).

### Stepwise regression analysis and results

To test hypothesis 3, the moderating effect of *“eaud”* on the impact of *“rjgdp”* and *“eqi,”* i.e., on the basis of economic development, environmental auditing is more likely to promote the improvement of ecological and environmental quality. In this study, the multivariate stepwise hierarchical regression method (Stepwise) was used to examine the validation, the valid sample size of the questionnaire was 217, and the results of the regression analysis are shown in Table 7.

**Table 7:** Stepwise regression analysis results of environmental audit on the relationship between economic development and ecological environment quality.

Dependent variable → independent variable ↓	*β*	*eqi*			
		Model 1	Model 2	Model 3	Model 4
Step 1: control variables					
1. Gender		0.041	0.019	0.038	0.029
2. Nature of work unit		−0.255^***^	−0.102^**^	−0.071^**^	−0.076^**^
3. Education		0.030	−0.023	−0.111^***^	−0.101^**^
4. Major		0.297^***^	0.070^*^	0.048	0.041
Step 2: Explanatory variables					
5. rjgdp			0.732^***^	0.193^***^	0.196^***^
Step 3: moderating variables					
6. eaud				0.696^***^	0.686^***^
Step 4: Moderating variables * explanatory variables					
7. rjgdp﹡eaud					0.067^**^
F-value		9.262	65.885	118.596	103.616
P-value		0.000	0.000	0.000	0.000
R^2^ value		0.149	0.610	0.766	0.776
ΔR^2^ value		0.149	0.461	0.156	0.010
VIF value		1.079	1.163	2.984	1.070
D-W value		1.523	1.936	1.803	1.769

^*^p < 0.1; ^**^p < 0.05; and ^***^p < 0.01.

Table 7 above reports the results of microscopic survey data and stepwise hierarchical regression. The last two rows of the table reflect the VIF factor and D-W index tests of the variables in each model. It is clear from the table that all the index values are within the standard control values and the results are satisfactory, indicating that there is no multicollinearity in the models and no autocorrelation between the residuals of the variables. The significance levels of all R^2^ *F*-values are below 0.001, indicating that the overall effect of all regression models is relatively satisfactory and suitable for causality testing.

In the first step, the control variables, i.e., individual characteristics variables, were put in to try to eliminate the influence of some disturbances, and the control variables explained a total of 14.9% (*F* = 9.262, *p* < 0.001) of the variance of ecological environment quality (*eqi*). Since individual characteristics often reflect the influence of multiple factors at the same time, the meaning of regression coefficients of individual characteristics variables is not derived in this paper.

In the second step, the explanatory variable, namely economic development (*rjgdp*), was put into the regression equation. Results Model 2 shows that there is a significant positive causal relationship between economic development (rjgdp) and ecological environment quality (eqi) at the level of 1% (β = 0.732, *p* < 0.01), The resulting model increased the explanatory power of the variance of ecological environmental quality (*eqi*) by 41.6% (*F* = 65.855, *p* < 0.01), which indicates that the level of economic development (*rjgdp*) has a significant cause explanatory power on the improvement of ecological environmental quality (*eqi*), which further supports the previous model hypothesis 1, that economic development has a significant effect on the improvement of ecological quality, all else being equal.

In order to filter out the possible power of environmental audit (*eaud*) itself on the improvement of ecological environment quality (*eqi*) before testing the moderating effect, this paper put environmental audit (*eaud*) into the regression equation in the third step, Results Model 3 found that environmental audit (*eaud*) had a significant causal relationship with ecological environment quality (*eqi*) at the level of 1%(β = 0.696, *p* < 0.01), and found that the explanatory power of environmental audit (*eaud*) variable on the improvement of ecological environment quality (*eqi*) increased by 15.6% (*F* = 118.596, p < 0.01), indicating that environmental audit (*eaud*) by itself has a 15.6% degree of influence on the relationship between economic development level (*rjgdp*) and ecological environmental quality (*eqi*), which also further supports the previous model hypothesis 2, that environmental audit has a significant causal influence on the improvement of ecological environmental quality, all else being equal.

To verify whether there is a moderating effect of environmental audit (*eaud*) between the level of economic development (*rjgdp*) and ecological environmental quality (*eqi*), the fourth step puts the decentered interaction product term (rjgdp*eaud) of government environmental audit (*eaud*) and the level of economic development (*rjgdp*) into the regression equation, The results show that the entry of these variables makes rjgdp*eaud in model 4 have a significant causal relationship with ecological environment quality (*eqi*) at the level of 1%(β = 0.067, *p* < 0.01), and it is found that the entry of this set of variables increases the model’s explanatory power of environmental audit (*eaud*) and ecological environment quality (*eqi*) by 1.0% (*F* = 103.616, *p* < 0.05), i.e., the decentered interaction product term (rjgdp*eaud) of environmental audit (*eaud*) and economic development level (*rjgdp*), in addition to excluding the effects of various other variables and their own existence, can also have a significant effect on ecological environmental quality (*eqi*) variance by a significant increase of 1.0% in explanatory power, indicating a moderating effect of environmental audits (*eaud*), which verifies the previous model hypothesis 4, i.e., the moderating effect of environmental audits on the impact of economic development and ecological environment.

**Figure 2:**
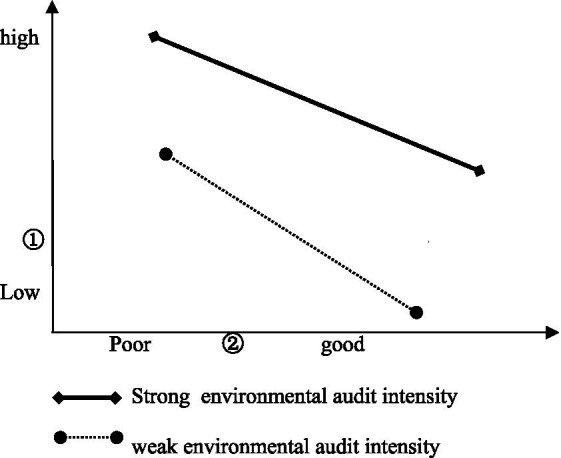
Economic development and ecological environment: the moderating effect of environmental audits. ① represents the level of economic development; ② represents the quality of ecological environment.

The regression coefficient β = 0.067 for the interaction product of economic development level and decentered environmental audits was found to be positive, indicating that environmental audits play a positive moderating effect in the relationship between economic development level and ecological environment quality, implying that the higher the economic development level, the better the ecological environment quality improvement for those regions with more environmental audits. On the contrary, for those regions with less environmental audits, the effect of ecological environment improvement is poorer (Figure 2).

## Conclusion

This paper empirically examines the relationship between economic development, environmental auditing, and ecological environment and their impacts using micro-survey data from 21 regions in Guangdong Province.

First, there is a significant positive correlation between economic development and ecological environment, and economic development can significantly contribute to the improvement of ecological environment quality. Therefore, governments at all levels can get rid of the difficult situation that economic development interferes with ecological and environmental protection work and achieve a win-win situation of economic growth and ecological and environmental quality improvement.

Second, there is a significant positive correlation between environmental auditing and ecological environment, and environmental auditing can help significantly improve the quality of ecological environment. Therefore, governments at all levels can play the functions of environmental auditing to monitor, warn, reveal, and correct deviations, and contribute to environmental governance with its unique characteristics of authority, independence, professionalism, comprehensiveness, and sensitivity to “cure existing diseases and prevent future diseases.” Then, in the process of promoting economic development, governments at all levels must increase the supply of environmental audits, optimize the allocation of audit resources, and promote full coverage of environmental audits to meet the current “double carbon” background of environmental auditing tasks and national requirements for full coverage of resource and environmental audits.

Third, environmental auditing has a positive moderating effect on economic development in improving ecological and environmental quality, and it is more significant on the basis of economic development. In the empirical test, it is also found that environmental auditing is a positive moderating effect in the relationship between the level of economic development and ecological environment quality, indicating that under the same level of economic development, for those regions where environmental auditing is carried out more intensely, it is more likely to improve ecological environment quality. Therefore, governments at all levels need to further follow the trend and actively carry out environmental audits to give full play to the important role of environmental audits and the active role of environmental governance subjects, so as to escort the realization of the “double carbon” goal and ultimately promote regional high-quality development.

## Data availability statement

The original contributions presented in the study are included in the article/supplementary material, further inquiries can be directed to the corresponding author.

## Author contributions

MX contributed to all the work of this study, which includes the research plan, conduct, survey design, data collection, data analysis, and report writing.

## Funding

This work was supported by Guangdong Province Education Science Planning Project (2019GXJK068); Huizhou University High-level Research Project (hzu201903); supported by Program for Innovative Research Team of Huizhou University; and the 2022 Huizhou Philosophy and Social Science Planning Project “Research on the Time Effect of Environmental Audit on Environmental Pollution Control under the Signaling Mechanism—Guangdong Province as an example.”

## Conflict of interest

The author declares that the research was conducted in the absence of any commercial or financial relationships that could be construed as a potential conflict of interest.

## Publisher’s note

All claims expressed in this article are solely those of the authors and do not necessarily represent those of their affiliated organizations, or those of the publisher, the editors and the reviewers. Any product that may be evaluated in this article, or claim that may be made by its manufacturer, is not guaranteed or endorsed by the publisher.

## Footnotes

^1^In 2020, the Chinese government officially proposed that the country will strive to reach the carbon peak by 2030 and achieve carbon neutrality by 2060, the "3,060" double carbon goal.

## References

[ref1] Cai C. Zheng K. K. Chen Y. (2019). Research on the influence of government environmental audit on corporate environmental responsibility information disclosure: based on the empirical evidence of environmental audit of "three Rivers and three lakes". Audit Res. 06, 3–12.

[ref2] Cai C. Zheng K. K. Wang P. (2021). Research on the impact of government environmental audit on enterprise environmental governance. Audit Res. 04, 3–13.

[ref3] Chen X. H. Xi Y. J. Wang Y. W. (2022). The results and enlightenment of the tenth WGEA global environmental audit survey. Audit Observ. 04, 50–55.

[ref4] Davis F. D. (1989). Perceived usefulness, perceived ease of use, and user acceptance of information technology. MIS Q. 13, 319–340. doi: 10.2307/249008, PMID: 24248003

[ref5] Ding S. H. Hu J. (2022). Environmental audit, environmental information disclosure and corporate environmental performance. Ecol. Econ. 38, 162–168.

[ref6] Li X. K. Wei J. (2010). Research on the decoupling of environmental pressure and economic development in Chongqing metropolitan area. J. Nat. Res. 25, 139–147.

[ref7] Liu S. H. (1997). Several issues about sustainable development and sustainable economic development. Contemp. Finan. Econ. 06, 15–18.

[ref8] Liu J. Y. (2015). National Audit in the process of National Governance Modernization: institutional guarantee and practical logic. Chin. Soc. Sci. 09, 64–83.

[ref9] Mishan E. J. (1967). The costs of economic growth. London: Staples Press London.

[ref10] Parry I. W. H. (1998). Pollution regulation and the efficiency gains from technological innovation. J. Regul. Econ. 3, 229–254.

[ref11] Peng Z. Liu X. Zhang W. (2020). Advances in the application, toxicity and degradation of carbon nanomaterials in environment: a review. Environ. Int. 134:105298. doi: 10.1016/j.envint.2019.105298, PMID: 31765863

[ref12] Prajogo D. Castka P. Yiu D. Yeung A. C. L. Lai K. H. (2016). Environmental audits and third party certification of management practices: Firms' motives, audit orientations, and satisfaction with certification. Int. J. Audit. 20, 202–210. doi: 10.1111/ijau.12068

[ref13] Ren X. S. Ma Q. Liu Y. J. Zhao G. H. (2021). The impact of carbon trading policies on industrial carbon productivity and the transmission mechanism. China Environ. Sci. 41, 5427–5437.

[ref100] Shen X. Z. (2005). A comparative study of R & D statistics and accounting. Educ. stat. 3, 22–24.

[ref14] Shi T. Yang S. Zhang W. (2020). Coupling coordination degree measurement and spatiotemporal heterogeneity between economic development and ecological environment--empirical evidence from tropical and subtropical regions of China. J. Clean. Prod. 244:118739. doi: 10.1016/j.jclepro.2019.118739

[ref15] Stafford S. L. (2006). State adoption of environmental audit initiatives. Contemp. Econ. Policy 24, 172–187. doi: 10.1093/cep/byj010

[ref16] Wang D. N. (2011). Strengthen government resource and environmental audit to promote transformation of economic development mode. Audit Res. 05, 880–888.

[ref17] Wang L. X. Liu S. B. (2017). Economic growth, urbanization and environmental pollution: an empirical analysis based on spatial simultaneous equations. South. Econ. 10, 126–140.

[ref18] Wang A. G. Zhang Z. (2019). Theoretical discussion on environmental audit service ecological civilization construction. Audit Res. 02, 43–47.

[ref19] Wu X. Guo J. J. (2019). The development status and enlightenment of foreign government environmental auditing—based on the WGEA global environmental audit survey. Audit Res. 01, 31–40.

[ref20] Wu D. Wu R. H. (2011). VAR model analysis of the relationship between economic growth and environmental pollution in different regions: an empirical study based on Guangzhou, Foshan and Zhaoqing economic circles. J. Environ. Sci. 31, 880–888.

[ref21] Yu L. C. Zhang W. G. Bi Q. Dong J. T. (2020). Will government environmental audits improve corporate environmental performance? Audit. Econ. Res. 35, 41–50.

[ref22] Zhang Q. Liang S. X. (2020). An empirical study on the relationship between foreign trade, economic growth and pollutant emissions—based on China's provincial panel data. Econ. Perspect. 01, 84–93.

[ref23] Zhao J. Li Y. Dang X. H. (2019). The impact of China's economic growth on environmental pollution: a spatial panel analysis of provincial data based on three types of pollutants. Urban Issues 08, 13–23.

[ref24] Zhao C. H. Rong X. (2022). The causes of regional environmental audit differences and the ways to close them. Audit Res. 03, 32–39.

[ref25] Zhou X. (2011). Path selection of environmental audit based on economic responsibility—analysis of environmental responsibility audit in economic responsibility audit. Audit. Res. 05, 24–27.

[ref26] Zhu W. P. Chen L. (2011). Research on the connotation and model of industrial upgrading—taking Guangdong’s industrial upgrading as an example. De Economist 02, 60–66.

